# Diagnosis, Treatment, and Prognosis of Patients with Primary Familial Gastrointestinal Stromal Tumor: A Case Report and Literature Review

**DOI:** 10.1093/oncolo/oyad168

**Published:** 2023-06-13

**Authors:** Miao Yan, Jianghua Lin, Man Shu, Yanji Luo, Kaiyu Sun, Shaohua Yang, Xinhua Zhang

**Affiliations:** Department of Gastrointestinal Surgery, The First Affiliated Hospital of Sun Yat-Sen University, Guangzhou, People’s Republic of China; Zhongshan School of Medicine, Sun Yat-Sen University, Guangzhou, People's Republic of China; Department of Pathology, The First Affiliated Hospital of Sun Yat-Sen University, Guangzhou, People's Republic of China; Department of Radiology, The First Affiliated Hospital of Sun Yat-Sen University, Guangzhou, People's Republic of China; Department of Gastrointestinal Surgery, The First Affiliated Hospital of Sun Yat-Sen University, Guangzhou, People’s Republic of China; Center of Digestive Disease, The Seventh Affiliated Hospital of Sun Yat-Sen University, Shenzhen, People's Republic of China; Department of Gastrointestinal Surgery, The First Affiliated Hospital of Sun Yat-Sen University, Guangzhou, People’s Republic of China

**Keywords:** gastrointestinal stromal tumors, familial GIST, KIT, germline mutation, p. W557R

## Abstract

Gastrointestinal stromal tumors are the most common mesenchymal tumors of the digestive tract, most of which are sporadic, and familial GISTs with germline mutations are rarely seen. Here, we report a 26-year-old female with a germline p. W557R mutation in exon 11 of the KIT gene. The proband and her father and sister presented with multifocal GIST and pigmented nevi. All 3 patients underwent surgery and imatinib therapy. To date, only 49 kindreds with germline KIT mutations and 6 kindreds with germline PDGFRA mutations have been reported. Summarizing the reported kindreds, the majority of familial GISTs manifest as multiple primary GISTs complicated with special clinical manifestations, including cutaneous hyperpigmentation, dysphagia, mastocytosis, inflammatory fibrous polyps, and large hands. Familial GISTs are generally thought to exhibit TKI sensitivity similar to that of sporadic GISTs with the same mutation.

## Teaser

Primary familial gastrointestinal tumors (GIST) are rare. This review summarizes the clinical features, treatment, and prognosis of the disease, aiming to help clinicians better diagnose and treat familial GIST.

**Implications for Practice**

Primary familial GIST is a very rare disease. So far, there is no consensus on the diagnosis, treatment, and prognosis of familial GIST. The authors review 55 previously reported primary familial GIST kindreds and summarize the clinical features, treatment, and prognosis of primary familial GIST. The authors hope this article will help clinicians better diagnose and treat familial GIST.

## Introduction

**Figure 1.**
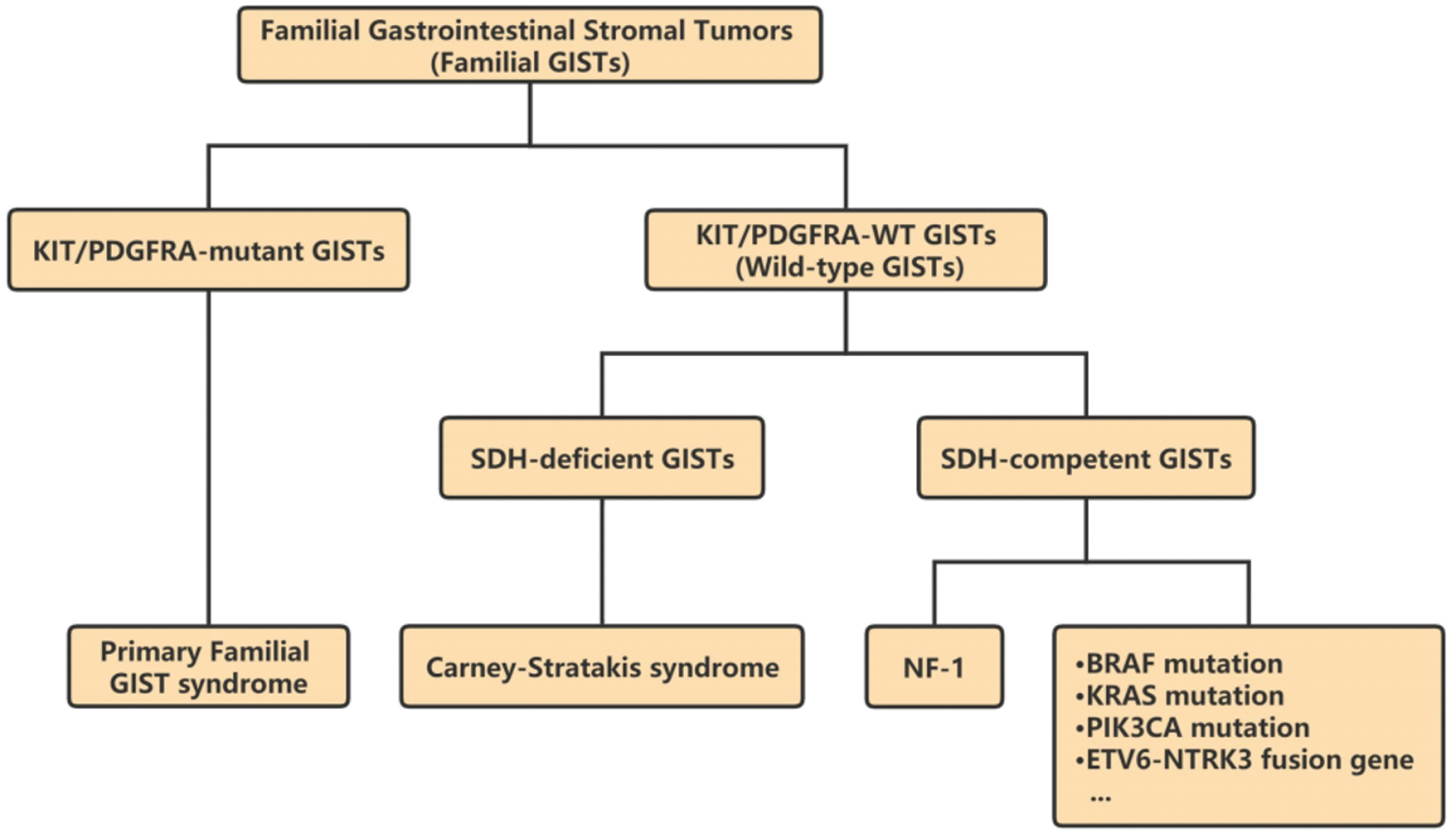
Classification of Familial Gastrointestinal Stromal tumors based on genetic and epigenetic background.

Gastrointestinal stromal tumors (GISTs) are the most common mesenchymal tumors of the gastrointestinal tract, originating from the interstitial cells of Cajal (ICCs) from the gastrointestinal tract and occurring in 10 per million people annually.^1^ The median age at detection of GISTs is 65 years, with no significant difference in incidence between males and females. GISTs can occur anywhere in the digestive tract, most commonly in the stomach or small intestine.^2^ Most GISTs (97%) are sporadic, and only a few cases have germline mutations, such as primary familial GIST syndrome, Carney–Stratakis syndrome, and neurofibromatosis type 1^3,4^ (Fig. 1). Familial GISTs are rare autosomal dominant disorders. Compared with sporadic GISTs, they often present with multifocal tumors and early onset (40-50 years old).^5^ In addition to GISTs, patients with germline mutations may have some special clinical manifestations, such as cutaneous hyperpigmentation, dysphagia, mastocytosis, large hands, and inflammatory fibrous polyps.^4^

**Figure 2.**
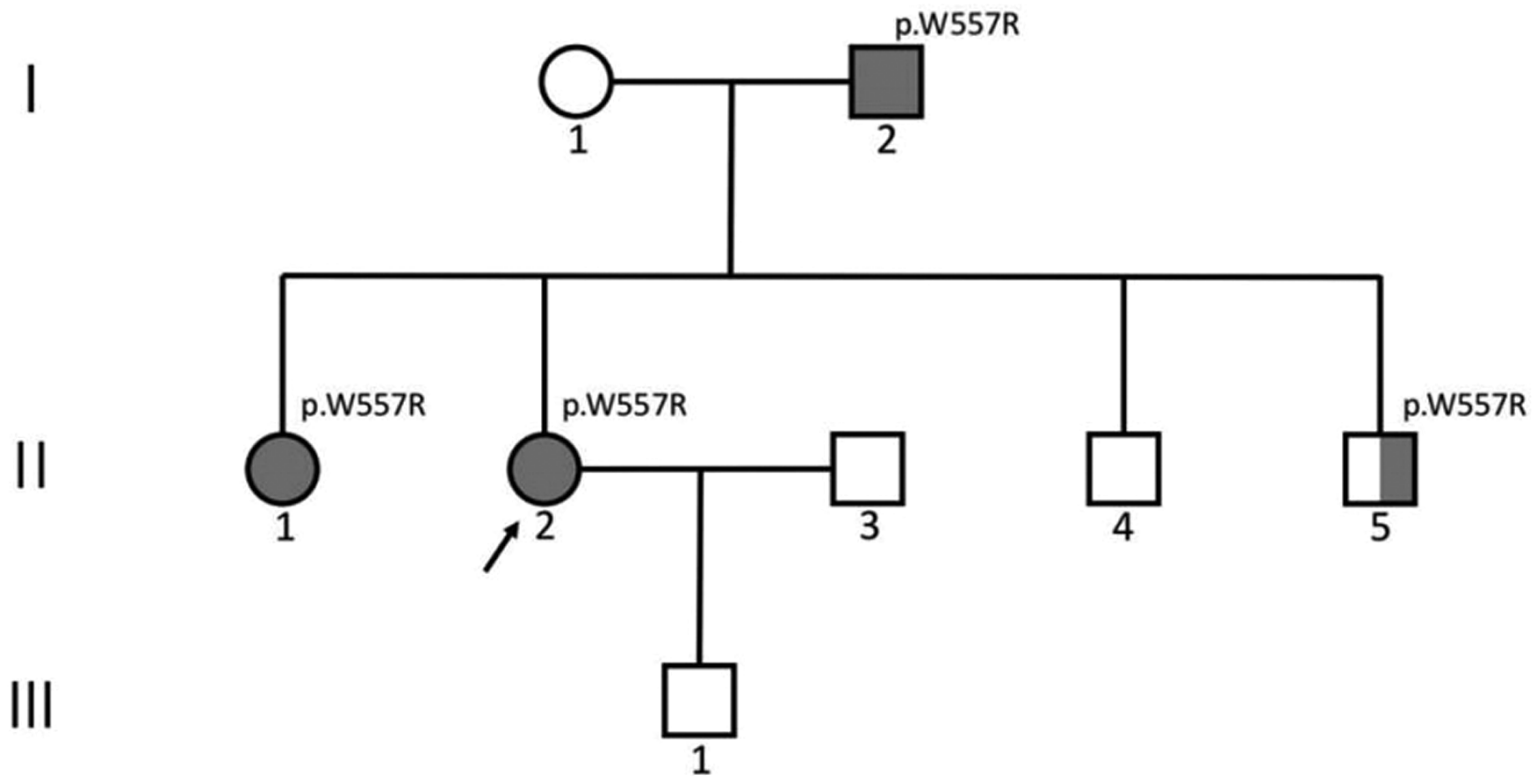
Pedigree of the family with germline KIT mutation in exon 11 (p.W557R). Squares, male; circles, females; solid symbols, family members with multiple GISTs and pigmented nevi; half-solid symbols, family members with pigmented nevi.

Familial GISTs with germline KIT or PDGFRA mutations are rare, with only 49 kindreds with germline KIT mutations and 6 kindreds with germline PDGFRA mutations reported to date (Supplementary Table S1). In this paper, we describe a 26-year-old female (II-2) with multiple GISTs and pigmented nevi with a KIT germline mutation in exon 11 (p.W557R). After resection of multiple tumors and part of the small intestine, she received imatinib for adjuvant therapy. Remarkably, the proband’s father (I-2) and sister (II-1) both had similar multifocal GISTs and pigmented nevi with germline KIT mutation in exon 11 (p.W557R) (Fig. 2). Both underwent surgery and imatinib therapy. One of the proband’s brothers (II-5) also developed pigmented nevi and was found to have germline KIT mutation in exon 11 (p. W557R), even though no evidence of GIST had yet been found (Fig. 4c). We also reviewed the relevant literature to better understand the clinical and histopathological characteristics and treatment of familial GISTs.

**Figure 3.**
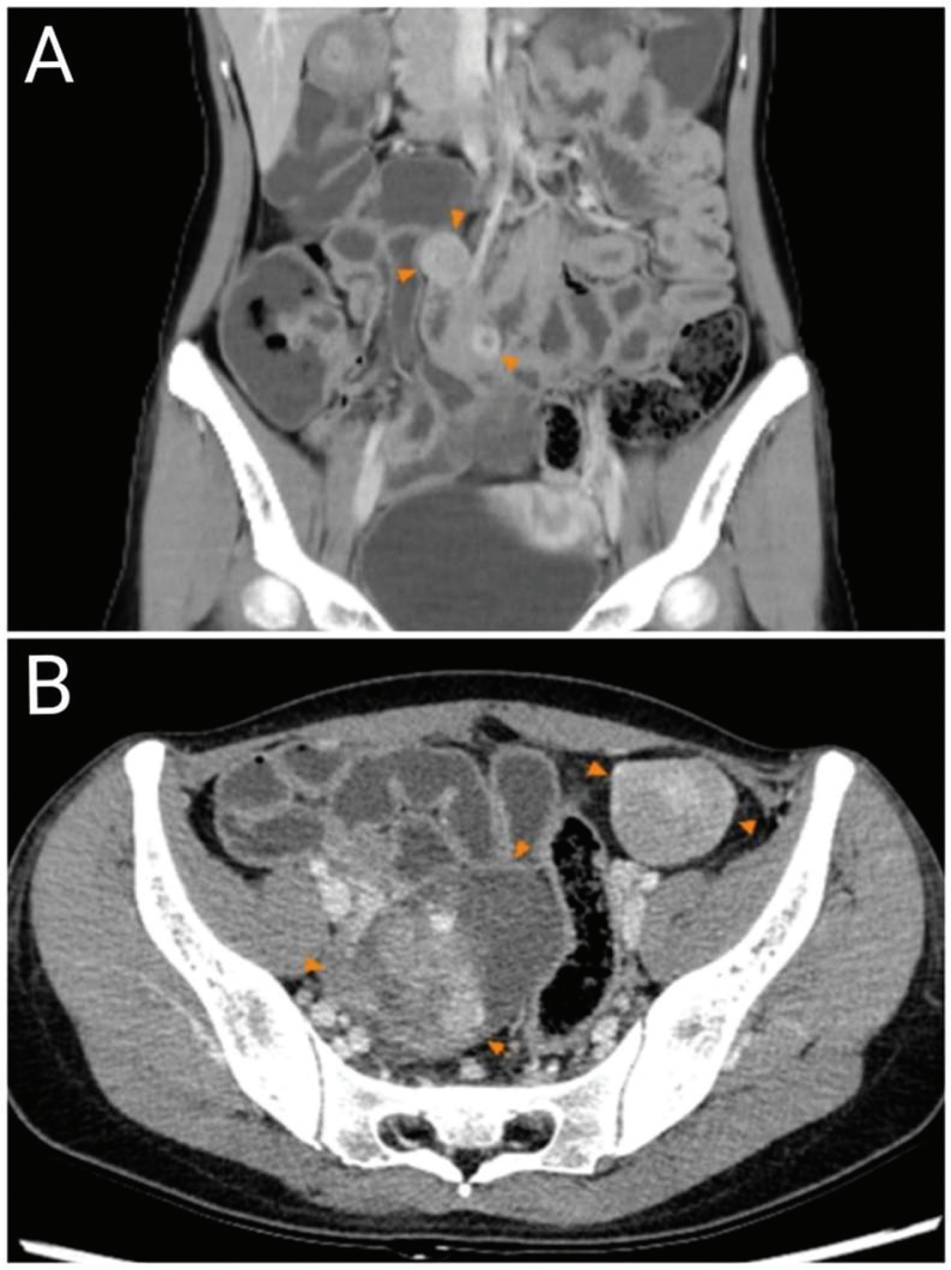
The imaging results of contrast-enhanced CT of the abdomen: (**A**) The arrows point to 2 tumors in the lower abdomen with diameters of 19 and 16 mm. (**B**) The arrows point to a mass of approximately 66 mm × 49 mm × 54 mm on the right side of the uterus and a mass of approximately 37 mm × 32 mm next to the sigmoid colon.

**Figure 4.**
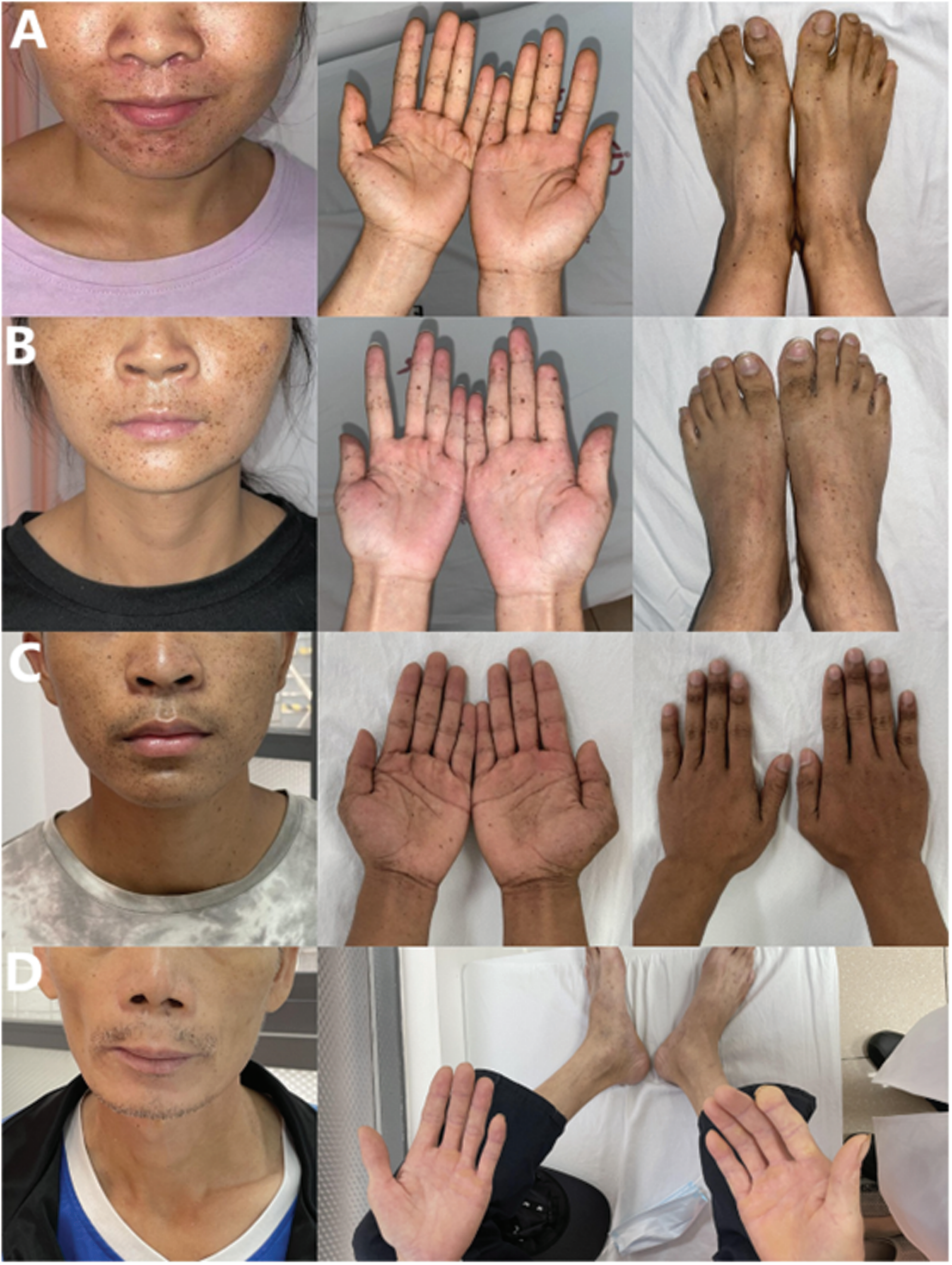
(**A**) The proband (II-2) had a significant pigmented nevus on the face and limbs prior to the initiation of imatinib. (**B**) The proband’s sister (II-1) had an increase in pigmented nevus on the face and limbs after discontinuation of imatinib. (**C**) The proband’s brother (II-5) also had a significant pigmented nevus on his face and limbs. (**D**) After a period of treatment with imatinib, the proband’s father (I-2) had fewer pigment nevi on his face and limbs, and his hair color and complexion turned white.

## Case Presentation

In 2022, a 26-year-old woman (II-2) with a 6-month history of postpartum dizziness and fatigue underwent routine transvaginal ultrasound after delivery showing enlargement of the right ovaries. After 3 months of observation, a follow-up transvaginal ultrasound suggested a solid mass in the upper left and right region of the uterus and abdominal cavity, considering the possibility of peritoneal-origin tumors. Computed tomography (CT) revealed multiple nodules and masses (measuring 19-66 mm in diameter) in the pelvis and lower abdomen, closely related to adjacent intestinal walls (Fig. 3). Physical examination revealed that the proband’s face, trunk, and limbs had pigmented nevi of varying sizes, clear boundaries, and smooth surfaces (Fig. 4a), and there were no gastrointestinal signs or symptoms.

**Figure 5:**
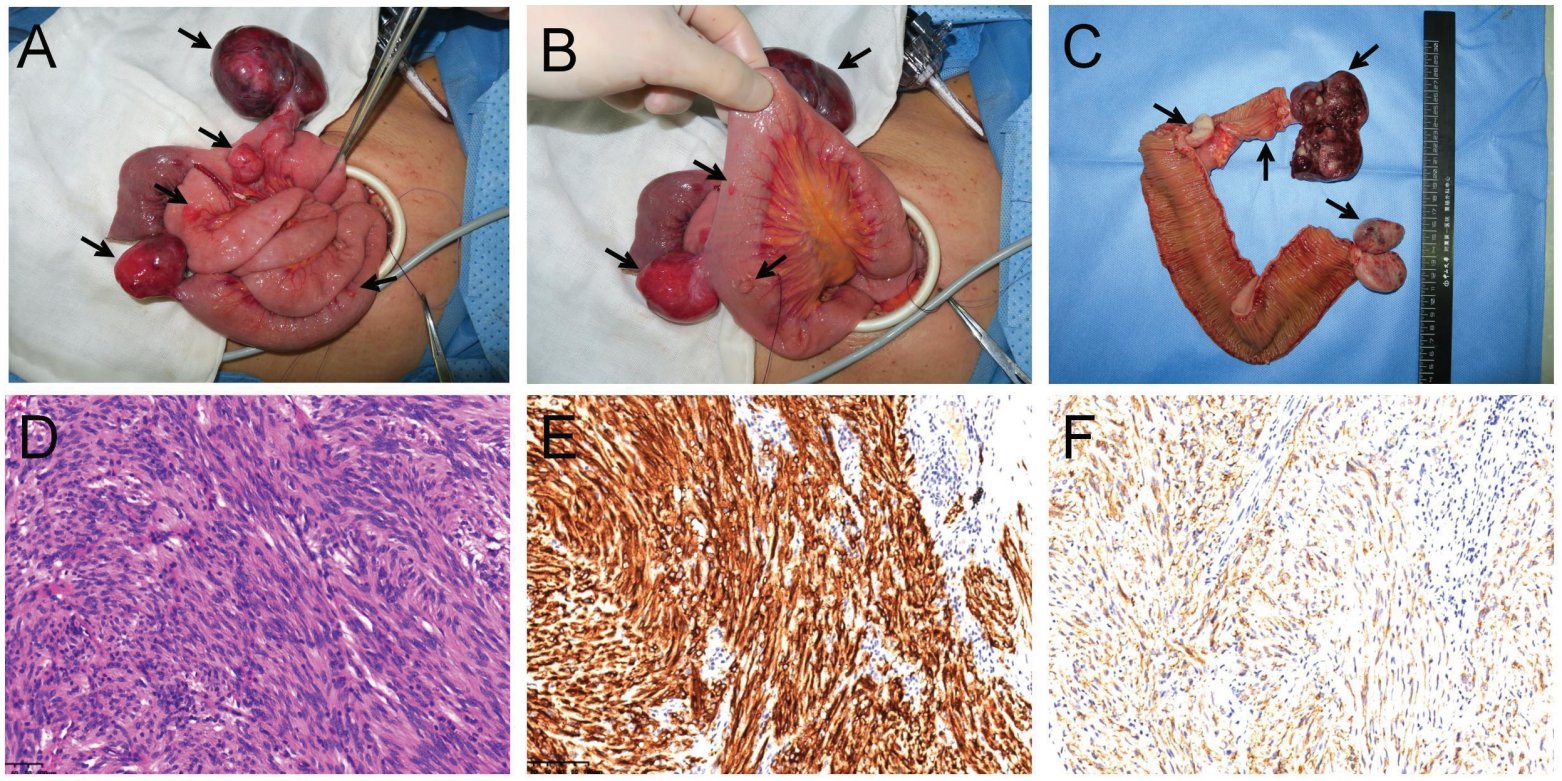
(**A**-**C**) Proximal jejunum and tumors in the 26-year-old patient. The intestinal tube was 11 cm long, 2.5 cm in diameter, and 0.6 cm in wall thickness, and multiple tumor nodules could be seen on the serous surface, of which the largest tumor was approximately 6.5 × 4.5 × 4 cm. (**D**) Haematoxylin and eosin (H&E) staining of part of the jejunum with the exophytic growing GIST (original magnification: ×20). (**E**) Immunohistochemical staining of CD117 (original magnification: ×20). (**F**) Immunohistochemical staining of DOG1 (original magnification: ×20).

According to typical clinical manifestations and family history, the proband was considered to have multiple primary familial GISTs. We performed laparoscopic resection of multifocal tumors of the small intestine. During the operation, 10 tumors of various sizes (1.3 to 6.5 cm) were found on the serous surface of the jejunum from 8 to 120 cm from the ligament of Treitz (Fig. 5a, 5b, 5c). Lymph nodes of the mesojejunum were soft and enlarged. All the tumors and parts of the small intestine were removed, as well as parts of the enlarged lymph nodes.

Histopathological examination of samples from the jejunum and ileum revealed CD117, DOG-1, and SDHB-positive spindle cells and partial CD34-positive spindle cells, and the Ki-67 index was 2%. The mitotic rate was 1 per 5 mm^2^ (Fig. 5d, 5e, 5f). Multifocal GISTs of high risk were diagnosed according to the NIH 2008 Revised GIST Hazard Grading System.

We conducted a detailed investigation of the proband’s family history. Her father (I-2) and elder sister (II-1) also had a history of multifocal GISTs and similar pigmented nevi. Her sister was diagnosed with multifocal GISTs in the small intestine in 2015 at the age of 23. She had been admitted to a local hospital for dark stools, while capsule endoscopy found masses in the upper segment of the small intestine. She underwent resection of GISTs (measuring from 3 to 10 cm) and part of the small intestine, and pathological diagnosis revealed multifocal GISTs. The majority of tumor cells were positive for CD117, DOG-1, and CD34, and the Ki-67 index was 8%. The mitosis rate was less than 5/50 HPF. She had received imatinib 600 mg QD as adjuvant therapy until April 2019 and abdominal enhanced CT annually for regular follow-up. The latest (2022-03) CT suggested no signs of tumor recurrence. After a period of imatinib use, her partial hair and complexion turned white, and the number of pigmented nevi on her body diminished, accompanied by a certain degree of hair loss. However, after stopping the drug, her hair gradually returned to black, and the number of pigmented nevi on her body also increased (Fig. 4b).

The proband’s father (I-2) underwent distal gastrectomy at a local hospital in 2002 (at the age of 25) for “upper gastrointestinal haemorrhage.” In November 2020 (at the age of 53), he went to the emergency department of a local hospital with “abdominal pain” and was diagnosed with multifocal GISTs of the small intestine with abdominal metastasis and tumor necrosis after emergency laparotomy and tumor resection. The local hospital performed debulking surgery to relieve symptoms (R2). Postoperative pathology revealed multiple GISTs of high risk (NIH 2008 Revised GIST Hazard Grading System), and tumor cells were positive for CD117, DOG-1, and SMA; partially positive for CD34; and the Ki-67 index was 2%. He began taking imatinib 400 mg QD regularly after resection. Tumor progression was confirmed 9 months after the operation, but he still took imatinib at the same dose due to slow tumor growth. In August 2022, he was referred to our hospital, and abdominal enhanced CT showed multiple abdominal nodules. Due to financial constraints, he declined the suggestion of surgery or second-line targeted therapy. After taking imatinib for a period of time, he also showed hair and complexion changes similar to his daughter, his hair and complexion turned white, and the number of pigmented nevi on the body decreased (Fig. 4d). However, he did not experience hair loss.

Considering the rarity, typical family history, multifocality, early onset and special pigmented nevi, we recommend that patients undergo genetic analysis to explore germline mutations. Genetic analysis revealed a pathogenic germline mutation in exon 11 of KIT (c.1669 T > C, p.W557R). We conducted genetic testing on patients’ relatives. Among the first-degree relatives, the proband’s sister (II-1), father (I-2) and younger brother (II-5) also had the same germline mutation, while another brother of the proband (II-4) did not. The son of the proband (III-1) failed to obtain genetic testing results due to unqualified specimens. Only 2 of the proband’s second-degree relatives (2 uncles) were willing to undergo genetic testing, and no pathogenic germline mutations in the KIT gene were found. The remaining second-degree relatives did not have abdominal tumors or pigmented nevi.

## Discussion

Activating mutations in KIT and PDGFRA are considered the main oncogenic drivers of GIST. KIT is a member of the type III receptor tyrosine kinase family, while PDGFRA is a homolog of KIT, both of which have similar structures and downstream signaling pathways.^6^ Approximately 75% of GISTs have KIT mutations, with the most common mutation occurring in exon 11, followed by 9, 13, and 17. PDGFRA mutations are present in 10%-15% of GISTs, most frequently occurring in exons 12, 14, and 18. The remaining GISTs (approximately 5%-10%) have other genetic alterations involving SDH complex genes, NF-1, and BRAF.^3^

The majority of GISTs (97%) are sporadic, and reports of familial GISTs with germline mutations are very rare. In total, 55 case reports of primary familial GISTs were retrieved from the PubMed database, 49 of which described hereditary GISTs due to germline KIT mutations and 6 of which described familial GISTs associated with germline PDGFRA mutations. Including the 3 patients mentioned in this article, there was a total of 112 patients (Table 1). Among them, there were more females than males (61 vs. 41, 54.46% vs. 36.61%, and 10 patients without gender mentioned). Among the 102 patients with age mentioned, the median age at diagnosis was 41.5 years (less than 65 years for sporadic GISTs), and 87 patients (77.68%) had multifocal GISTs at initial diagnosis, consistent with the characteristics of early onset age and multifocal tumors of familial GIST. Familial GIST tumors are mainly found in the stomach and small intestine. The size of the tumor was clearly mentioned in 54 patients (48.21%), of which the largest tumor diameter was 20 cm and the smallest less than 0.5 cm. Of the 37 patients who explicitly mentioned tumor status, 15 patients (40.54%) were initially diagnosed with metastasis. Seventy-five patients (66.96%) had special clinical manifestations, including cutaneous hyperpigmentation (50, 66.67%), dysphagia (21, 28.00%), mastocytosis (7, 9.33%), inflammatory fibrous polyps (2, 2.67%), fibroids (3, 4.00%), gastrointestinal lipomas (2, 2.67%), and large hands (9, 12.00%). Treatment was explicitly mentioned in 90 patients (80.36%), of which 87 (77.68%) underwent surgery, tyrosine kinase inhibitor (TKI) or a combination of both.

**Table 1.** Review of primary familial GISTs.

		Number of patients	Percentage
Mutated genes	Kit	100	89.29
	PDGFRA	12	10.71
Gender	Male	41	36.61
	Female	61	54.46
	Not mentioned	10	8.93
Age at diagnosis	≤20 yr	5	4.46
	>20 yr, ≤40 yr	43	38.39
	>40 yr, ≤60 yr	40	35.71
	>60 yr	14	12.50
	Not mentioned	10	8.93
Whether multifocal tumors	Yes	87	77.68
	No	7	6.25
	Not mentioned	18	16.07
Tumor location	Stomach	8	7.14
	Small intestine	30	26.79
	Stomach and small intestine	25	22.32
	Stomach, small intestine and other location^a^	14	12.50
	Other location	4	3.57
	Not mentioned	31	27.68
The maximum diameter of tumor	≤5 cm	24	21.43
	>5 cm, ≤10 cm	22	19.64
	>10 cm	8	7.14
	Not mentioned	58	51.79
Whether metastasis at diagnosis	Yes	15	13.39
	No	22	19.64
	Not mentioned	75	66.96
Whether non-tumor symptoms ^b^	Cutaneous hyperpigmentation	50	44.64
	Dysphagia	21	18.75
	Systemic mastocytosis	7	6.25
	Inflammatory fibroid polyps	2	1.79
	Fibroids	3	2.68
	Gastrointestinal lipomas	2	1.79
	Large hands	9	8.04
	No	34	30.36
	Other^c^	3	2.68
Treatment	Surgery	47	41.96
	TKI	11	9.82
	Surgery and TKI	29	25.89
	Other^d^	3	2.68
	Not mentioned	22	19.64

^a^Rectum, pelvis, liver, lungs, and peritoneum.

^b^Some patients have multiple non-tumor symptoms at the same time.

^c^Breast cancer, thyroid cancer.

^d^Chemotherapy.

Among the 50 kindreds with KIT germline mutations, germline mutations in exon 11 were most common (31, 62.00%), followed by exon 13 (10, 20.00%), exon 17 (5, 10.00%), exon 9 (2, 4.00%), and exon 8 (1, 2.00%). Among the 6 kindreds with PDGFRA germline mutations, kindreds with exon 12 germline mutations were the most common (3, 50%), followed by exon 18 (2, 33.33%) and exon 14 (1 16.67%). The special clinical manifestations of familial GISTs are related to the type of gene mutation (Supplementary Table S2).

### Mechanisms Underlying KIT and PDGFRA Mutations Leading to Tumorigenesis

KIT and platelet-derived growth factor receptor-α (PDGFRA) are members of the type III receptor tyrosine kinase family, both of which have similar structures. Binding of extracellular ligands (stem cell factor and PDGFA) to the receptor causes dimerization of the receptor, leading to autophosphorylation of tyrosine residues, which in turn activates downstream signaling pathways.^7^ Oncogenic mutations of KIT or PDGFRA can lead to ligand-independent receptor activation, thereby activating downstream signaling pathways and leading to uncontrolled cell proliferation and differentiation.

### Special Clinical Manifestations and Pathological Features of Primary Familial GIST

Remarkably, in addition to GISTs, primary familial GISTs often have some special clinical manifestations. GISTs bearing KIT exon 11 germline mutations are likely to have cutaneous hyperpigmentation or pigmented nevi. Patients with KIT exon 13 or 17 germline mutations are at risk for dysphagia. Patients with PDGFRA germline mutations often have large hands or inflammatory fibroid polyps. We described a 26-year-old woman with KIT exon 11 p. W557R germline mutation who, along with 2 family members, presented with multifocal GIST and pigmented nevi.

Multiple cases have been reported in which GISTs with KIT germline mutations are complicated with cutaneous hyperpigmentation. After using imatinib for a period of time, some patients developed gray hair and pale skin and had reduced skin pigment spots.^8-11^ In this paper, the proband’s father (I-2) and sister (II-1) experienced graying of hair and skin tone and had reduced skin pigment spots after receiving prolonged imatinib. The KIT gene plays an important role in the development of ICCs, pigment cells, and mast cells.^12,13^ As a tyrosine kinase inhibitor, the inhibitory effect of imatinib on c-KIT is likely to lead to inhibition downstream of the promoter of the tyrosinase gene, thereby inhibiting the production of pigment and resulting in changes in hair color and skin tone.^14^ The proband’s sister (II-1) experienced slight alopecia after using imatinib, which was considered an adverse reaction of imatinib.

Primary familial GIST is not different from sporadic GIST in histopathology, with 3 types of cellular morphology: spindle cell type, epithelioid cell type, and mixed type. Immunohistochemistry is often positive for CD117 and DOG-1.

### Treatment of Primary Familial GIST

A strategy combining surgery and imatinib may be the best treatment to guarantee a better prognosis for patients.^15^ Primary familial GISTs usually present as multifocal tumors that may not be resectable by R0 surgery. Patients are likely to develop more tumors during their lifetime; thus, it is uncertain whether resection of small, low-grade tumors improves long-term survival. Bachet et al suggested that surgery should be considered in the following situations: acute complications, GIST with maximum diameter ≥ 5 cm, tumor symptoms or tumor growth despite imatinib treatment.^16^

Familial GISTs are generally thought to exhibit TKI sensitivity similar to that of sporadic GISTs with the same mutation.^17^ In sporadic GISTs, GISTs with KIT exon 11 mutations are sensitive to imatinib.^18,19^ The proband’s father received continued imatinib after postoperative recurrence, and the tumor was generally stable. A recent study reported a germline mutation case of KIT exon 18 A829P with primary resistance to imatinib.^20^ In sporadic GIST, A829P is commonly found in secondary resistant mutations after imatinib treatment. These reports suggest that germline mutations in GISTs may share similar TKI sensitivity with somatic mutations. In this paper, after surgery, we decided to give the proband imatinib 400 mg daily for adjuvant therapy, considering the type of gene mutation and high-level risk classification.

Whether familial GIST should extend the duration of imatinib treatment remains controversial. This is mainly because there are too few relevant case reports and a lack of evidence. Regarding the similarities and differences between familial GIST and sporadic GIST of tumor biological behavior and natural course of the disease, the reported patients rarely have long-term follow-up records. Heinrich et al^18^ advocate the use of TKIs for long-term preventive treatment, or even life-long treatment, for patients with sensitive germline mutations. However, preclinical data from mouse models show that long-term use of TKIs has potential long-term effects on pregnancy and transplantation. We should carefully consider the long-term use of TKIs in younger patients and patients who need transplantation.^21^ In addition, given the indolent state of tumor progression of the proband’s father after the failure of first-line imatinib treatment, it is speculated that GIST with germline KIT exon 11 mutation may have a relatively inert biological behavior compared with sporadic cases.

GIST patients with germline mutations are advised to consult hematologists before initiating imatinib therapy to rule out the presence of mastocytosis. For carriers of pathogenic germline mutations who have fertility needs, prenatal genetic diagnosis, or assisted reproductive technology can be performed to reduce the possibility of passing the pathogenic mutation to their offspring.

### Screening and Follow-up of Primary Familial GIST

In the following situations, clinicians should be alert to the possibility of familial GIST: (1) age < 45 years at diagnosis; (2) multifocal GISTs; (3) first-degree relatives with GIST; (4) GIST with skin pigmentation changes, mastocytosis, large hands, and other non-tumor symptoms; and (5) diffuse proliferation of Cajal interstitial cells in the gastrointestinal tract wall at a distance from the tumor. Patients with the above conditions, especially those with any of the 2-5 manifestations, should undergo germline detection of KIT and PDGFRA genes.

For patients with confirmed primary familial GIST, regular follow-up visits are recommended at intervals similar to those for sporadic GISTs with corresponding malignant risk grades. For relatives carrying causative genes, we recommend initiating abdominal imaging at age 20 years or 2-5 years earlier than the youngest patient diagnosed in the family to detect GIST early.^22^ CT, especially contrast-enhanced CT, is the preferred imaging modality for GIST. MRI can be used as an alternative for long-term follow-up since it is nonradioactive. In this kindred, the earliest age at diagnosis is 23 years, and the proband’s brother (II-5) is 18 years old this year. Therefore, we recommend that he undergo regular abdominal imaging immediately.

All first-degree relatives of patients with familial GIST should seek genetic counselling and germline genetic testing to determine whether they carry causative germline mutations. Individuals younger than 18 years can wait for adulthood and provide informed consent before undergoing germline genetic testing. If relatives exhibit specific non-tumor symptoms at a younger age, germline genetic testing may be considered in advance. The NHS Genomics Service in the UK has extended germline testing criteria for KIT and other GIST predisposition genes to affected patients before the age of 50 years if they have associated mastocytosis, family history of GIST or associated cancers.

In addition, clinicians should be aware that patients with familial GISTs may have a higher risk of developing other tumors.^23^ In a case report published by Mara Fornasarig in 2020, in addition to GIST, the index case also developed breast cancer and thyroid papillary carcinoma.^10^ In the case reported by Wadt, the patient had breast cancer with a GIST.^24^ Whether long-term TKI therapy or frequent imaging would further increase the risk of second malignancy in this population remains to be determined. Clinicians should be alert to the possibility of a second malignancy during treatment.

## Conclusions

Familial GISTs carrying germline mutations are very rare and easily ignored clinically. However, with the maturity and popularization of genetic testing technology, familial GIST patients are increasingly being found. Clinicians should advise young patients and those with relevant family history to undergo genetic testing and watch out for specific clinical symptoms, such as cutaneous hyperpigmentation, dysphagia, mastocytosis, inflammatory fibrous polyps, and large hands. There are still no guidelines for the treatment, prognosis, or monitoring of familial GISTs, and we recommend more active treatment and follow-up strategies.

## Supplementary Material

oyad168_suppl_Supplementary_Table_S1

## Data Availability

No new data were generated or analyzed in support of this research.
