# Supplementary material for: Diagnosis, Treatment, and Prognosis of Patients with Primary Familial Gastrointestinal Stromal Tumor: A Case Report and Literature Review
Source: Oncologist. 2023 Jun 13;28(12):e1134–41. doi: 10.1093/oncolo/oyad168 (PMC10712720; doi:10.1093/oncolo/oyad168)
Supplement: oyad168_suppl_Supplementary_Table_S1 [file oyad168_suppl_supplementary_table_s1.docx]

**Supplemental Table S1.** Reported cases of primary familial GIST

| Gene | Exon | Mutational type | Kindred | | Clinical characteristics [References] |
| --- | --- | --- | --- | --- | --- |
| KIT | 8 | D419del | 1 | Multiple GISTs, Systemic mastocytosis, Dysphagia [1] | |
|  | 9 | K509I | 2 | Multiple GISTs, Systemic mastocytosis [2]; Multiple GISTs, Mastocytosis, Achalasia [3] | |
|  | 11 | Y533C | 1 | Multiple GISTs, ICC hyperplasia [4] | |
|  |  | W557R | 5 | Multiple gastrointestinal autonomic nerve tumour [5]; Multiple GISTs, Cutaneous hyperpigmentation [6]; Multiple GISTs [7]; Multiple GISTs, cutaneous hyperpigmentation [present report]; GIST, Cutaneous hyperpigmentation, Dysphagia [8] | |
|  |  | W557S | 1 | Multiple GISTs, generalized lentigines [9] | |
|  |  | W557L+K558E | 1 | Multiple GISTs, Hereditary breast cancer [10] | |
|  |  | V559A | 7 | Multiple GISTs, lentigines, cafe-au-lait macules [11]; Multiple GISTs, Cutaneous hyperpigmentation [12]; Multiple GISTs, melanosis, lentiginosis, Dysphagia [13]; Multiple GISTs, Cutaneous hyperpigmentation [14]; Multiple GISTs, Gastric malignant submucosal tumour, Cutaneous hyperpigmentation [15]; Multiple GISTs [16, 17] | |
|  |  | V559_V560del | 1 | Multiple GISTs, Cutaneous hyperpigmentation [18] | |
|  |  | V560del | 1 | multiple GISTs [19] | |
|  |  | V560G | 2 | Multiple GISTs, Cutaneous hyperpigmentation [17, 20] | |
|  |  | Q575_P577delinsH | 1 | Rectal GIST [21] | |
|  |  | L576P | 2 | Multiple GISTs, lentigines, Achalasia-like stenosis [22]; Multiple GISTs [23] | |
|  |  | L576_P577insQL | 1 | Multiple GISTs, Cutaneous hyperpigmentation [24] | |
|  |  | D579del | 8 | Multiple GISTs, Cutaneous hyperpigmentation, Dysphagia [25]; GIST [26, 27]; Multiple GISTs [28, 29]; Multiple GIST, Junctional melanocytic nevus [30]; Multiple GISTs, Cutaneous hyperpigmentation [31] | |
|  | 13 | K642T | 1 | Multiple GISTs, Dysphagia [32] | |
|  |  | K642E | 8 | Multiple GISTs, Breast cancer [33]; Multiple GISTs, multiple lentigines, Dysphagia [34]; Multiple GISTs [34, 35]; Multiple GISTs including rectal GIST [36]; Multiple GISTs, Cutaneous hyperpigmentation [37]; multiple GISTs including rectal GIST, Cutaneous hyperpigmentation, Dysphagia [38] | |
|  |  | N655K | 1 | Multiple GISTs, lentigines, atypical junctional nevus, Breast and thyroid cancer [39] | |
|  | 17 | D820Y | 3 | Multiple GISTs, Dysphagia [40]; Multiple GISTs , Dysphagia [41]; Multiple GISTs including rectal GIST, Endometrial stromal sarcoma [42] | |
|  | 17 | D820G | 1 | Multiple GISTs [43] | |
|  | 17 | N822Y | 1 | Multiple GISTs [44] | |
|  | 18 | A829P | 1 | Multiple gastric subepithelial lesions, cutaneous hyperpigmentation, nevi [45] | |
| PDGFRA | 12 | Y555C | 1 | Multiple GISTs, Intestinal neurofibromatosis, Large hands [46] | |
|  | 12 | V561D | 2 | Multiple GISTs, Multiple fibrous tumours, lipoma [47]; Multiple GISTs, Multiple fibrous tumours, Lipoma [48] | |
|  | 14 | P653L | 1 | Multiple GISTs, Inflammatory fibroid polyps, Fibrous tumours [49] | |
|  | 18 | D846Y | 1 | Multiple GISTs, Large hands [50] | |
|  | 18 | D846V | 1 | Multiple GISTs, Multiple inflammatory fibroid polyps, Coarse skin, Large hands, Tooth loss [51] | |

*Modified from Fornasarig M, et al [39] with supplemented data.

**References:**

1. Hartmann K, Wardelmann E, Ma Y, Merkelbach Bruse S, Preussner LM, Woolery C, Baldus SE, Heinicke T, Thiele J, Buettner R, Longley BJ. Novel Germline Mutation of KIT Associated With Familial Gastrointestinal Stromal Tumors and Mastocytosis. GASTROENTEROLOGY. 2005 2005-01-01;129(3):1042-6.

2. Speight RA, Nicolle A, Needham SJ, Verrill MW, Bryon J, Panter S. Rare, germline mutation of KIT with imatinib-resistant multiple GI stromal tumors and mastocytosis. J CLIN ONCOL. [Case Reports; Journal Article]. 2013 2013-06-01;31(16):e245-7.

3. Halpern AL, Torphy RJ, McCarter MD, Sciotto CG, Glode LM, Robinson WA. A familial germline mutation in KIT associated with achalasia, mastocytosis and gastrointestinal stromal tumors shows response to kinase inhibitors. CANCER GENET-NY. [Journal Article]. 2019 2019-04-01;233-234:1-6.

4. Nakai M, Hashikura Y, Ohkouchi M, Yamamura M, Akiyama T, Shiba K, Kajimoto N, Tsukamoto Y, Hao H, Isozaki K, Hirai T, Hirota S. Characterization of novel germline c-kit gene mutation, KIT-Tyr553Cys, observed in a family with multiple gastrointestinal stromal tumors. LAB INVEST. 2012 2012-01-01;92(3):451-7.

5. Hirota S, Okazaki T, Kitamura Y, O'Brien P, Kapusta L, Dardick I. Cause of familial and multiple gastrointestinal autonomic nerve tumors with hyperplasia of interstitial cells of Cajal is germline mutation of the c-kit gene. AM J SURG PATHOL. [Letter]. 2000 2000-02-01;24(2):326-7.

6. Farag S, van der Kolk LE, van Boven HH, van Akkooi A, Beets GL, Wilmink JW, Steeghs N. Remarkable effects of imatinib in a family with young onset gastrointestinal stromal tumors and cutaneous hyperpigmentation associated with a germline KIT-Trp557Arg mutation: case report and literature overview. FAM CANCER. [Case Reports; Journal Article; Research Support, Non-U.S. Gov't; Review]. 2018 2018-04-01;17(2):247-53.

7. Antonescu CR, Viale A, Sarran L, Tschernyavsky SJ, Gonen M, Segal NH, Maki RG, Socci ND, DeMatteo RP, Besmer P. Gene expression in gastrointestinal stromal tumors is distinguished by KIT genotype and anatomic site. CLIN CANCER RES. [Journal Article; Research Support, Non-U.S. Gov't; Research Support, U.S. Gov't, P.H.S.]. 2004 2004-05-15;10(10):3282-90.

8. ROBSON ME, GLOGOWSKI E, SOMMER G, ANTONESCU CR, NAFA K, MAKI RG, ELLIS N, BESMER P, BRENNAN M, OFFIT K. Pleomorphic Characteristics of a Germ-Line KIT Mutation in a Large Kindred with Gastrointestinal Stromal Tumors, Hyperpigmentation, and Dysphagia. CLIN CANCER RES. 2004 2004-01-01;10(4):1250-4.

9. Hasegawa M, Shimizu A, Ieta K, Shibusawa K, Ishikawa O, Ishida-Yamamoto A, Tamura A. Generalized lentigines associated with familial gastrointestinal stromal tumors dramatically improved by imatinib treatment. J DERMATOL. [Case Reports; Letter]. 2020 2020-06-01;47(6):e241-2.

10. Sekido Y, Ohigashi S, Takahashi T, Hayashi N, Suzuki K, Hirota S. Familial Gastrointestinal Stromal Tumor with Germline KIT Mutations Accompanying Hereditary Breast and Ovarian Cancer Syndrome. ANTICANCER RES. [Case Reports; Journal Article]. 2017 2017-03-01;37(3):1425-31.

11. Gupta D, Chandrashekar L, Larizza L, Colombo EA, Fontana L, Gervasini C, Thappa DM, Rajappa M, Rajendiran KS, Sreenath GS, Kate V. Familial gastrointestinal stromal tumors, lentigines, and cafe-au-lait macules associated with germline c-kit mutation treated with imatinib. INT J DERMATOL. [Case Reports; Journal Article]. 2017 2017-02-01;56(2):195-201.

12. Maeyama H, Hidaka E, Ota H, Minami S, Kajiyama M, Kuraishi A, Mori H, Matsuda Y, Wada S, Sodeyama H, Nakata S, Kawamura N, Hata S, Watanabe M, Iijima Y, Katsuyama T. Familial gastrointestinal stromal tumor with hyperpigmentation: Association with a germline mutation of the c-kit gene. GASTROENTEROLOGY. 2001 2001-01-01;120(1):210-5.

13. Adela AS, Penaloza J, Gonzalez F, Abdo I, Rainville I, Root E, Carrero VR, Garber J. Dysphagia, melanosis, gastrointestinal stromal tumors and a germinal mutation of the KIT gene in an Argentine family. Acta Gastroenterol Latinoam. [Journal Article; Research Support, Non-U.S. Gov't]. 2014 2014-03-01;44(1):9-15.

14. Beghini A, Tibiletti M, Roversi G, Chiaravalli A, Serio G, Capella C, Larizza L. Germline mutation in the juxtamembrane domain of the kit gene in a family with gastrointestinal stromal tumors and urticaria pigmentosa. CANCER-AM CANCER SOC. 2001 2001-01-01;92(3):657-62.

15. Kuroda N, Tanida N, Hirota S, Daum O, Hes O, Michal M, Lee GH. Familial gastrointestinal stromal tumor with germ line mutation of the juxtamembrane domain of the KIT gene observed in relatively young women. ANN DIAGN PATHOL. [Case Reports; Journal Article]. 2011 2011-10-01;15(5):358-61.

16. Kim HJ, Lim SJ, Park K, Yuh YJ, Jang SJ, Choi J. Multiple gastrointestinal stromal tumors with a germline c-kit mutation. PATHOL INT. [Case Reports; Journal Article; Research Support, Non-U.S. Gov't]. 2005 2005-10-01;55(10):655-9.

17. KANG DY, PARK CK, KANG H, KIM K, CHOI JS, JIN SY, KIM HJ, JOO M, KANG MS, MOON WS, YUN KJ, YU ES. Multiple gastrointestinal stromal tumors : Clinicopathologic and genetic analysis of 12 patients. The American journal of surgical pathology. 2007 2007-01-01;31(2):224-32.

18. Nishida T, Hirota S, Taniguchi M, Hashimoto K, Isozaki K, Nakamura H, Kanakura Y, Tanaka T, Takabayashi A, Matsuda H, Kitamura Y. Familial gastrointestinal stromal tumours with germline mutation of the KIT gene. NAT GENET. 1998 1998-01-01;19(4):323-4.

19. Bamba S, Hirota S, Inatomi O, Ban H, Nishimura T, Shioya M, Imaeda H, Nishida A, Sasaki M, Murata S, Andoh A. Familial and multiple gastrointestinal stromal tumors with fair response to a half-dose of imatinib. INTERNAL MED. [Case Reports; Journal Article]. 2015 2015-01-20;54(7):759-64.

20. Yuan W, Huang W, Ren L, Xu C, Luan LJ, Huang J, Xue AW, Fang Y, Gao XD, Shen KT, Lv JH, Hou YY. Familial gastrointestinal stromal tumors with KIT germline mutation in a Chinese family: A case report. WORLD J CLIN CASES. [Case Reports]. 2022 2022-05-26;10(15):4878-85.

21. Wozniak A, Rutkowski P, Sciot R, Ruka W, Michej W, Debiec-Rychter M. Rectal gastrointestinal stromal tumors associated with a novel germline KIT mutation. INT J CANCER. [Case Reports; Journal Article; Research Support, Non-U.S. Gov't]. 2008 2008-05-01;122(9):2160-4.

22. Neuhann TM, Mansmann V, Merkelbach-Bruse S, Klink B, Hellinger A, Hoffkes HG, Wardelmann E, Schildhaus HU, Tinschert S. A novel germline KIT mutation (p.L576P) in a family presenting with juvenile onset of multiple gastrointestinal stromal tumors, skin hyperpigmentations, and esophageal stenosis. AM J SURG PATHOL. [Case Reports; Journal Article; Research Support, Non-U.S. Gov't]. 2013 2013-06-01;37(6):898-905.

23. Vale Rodrigues R, Santos F, Pereira Da Silva J, Francisco I, Claro I, Albuquerque C, Lemos MM, Limbert M, Dias Pereira A. A case of multiple gastrointestinal stromal tumors caused by a germline KIT gene mutation (p.Leu576Pro). FAM CANCER. [Case Reports; Journal Article]. 2017 2017-04-01;16(2):267-70.

24. Carballo M, Roig I, Aguilar F, Pol MA, Gamundi MJ, Hernan I, Martinez-Gimeno M. Novel c-KIT germline mutation in a family with gastrointestinal stromal tumors and cutaneous hyperpigmentation. American journal of medical genetics. Part A. 2005 2005-01-01;132A(4):361-4.

25. Forde PM, Cochran RL, Boikos SA, Zabransky DJ, Beaver JA, Meyer CF, Thornton KA, Montgomery EA, Lidor AO, Donehower RC, Park BH. Familial GI Stromal Tumor With Loss of Heterozygosity and Amplification of Mutant KIT. J CLIN ONCOL. [Case Reports; Journal Article]. 2016 2016-01-20;34(3):e13-6.

26. Tarn C, Merkel E, Canutescu AA, Shen W, Skorobogatko Y, Heslin MJ, Eisenberg B, Birbe R, Patchefsky A, Dunbrack R, Arnoletti JP, von Mehren M, Godwin AK. Analysis of KIT mutations in sporadic and familial gastrointestinal stromal tumors: therapeutic implications through protein modeling. CLIN CANCER RES. [Journal Article; Research Support, N.I.H., Extramural; Research Support, Non-U.S. Gov't; Research Support, U.S. Gov't, P.H.S.]. 2005 2005-05-15;11(10):3668-77.

27. Wali GN, Halliday D, Dua J, Ieremia E, McPherson T, Matin RN. Cutaneous hyperpigmentation and familial gastrointestinal stromal tumour associated with KIT mutation. CLIN EXP DERMATOL. 2019 2019-01-01;44(4):418-21.

28. Jones DH, Caracciolo JT, Hodul PJ, Strosberg JR, Coppola D, Bui MM. Familial gastrointestinal stromal tumor syndrome: report of 2 cases with KIT exon 11 mutation. CANCER CONTROL. [Case Reports; Journal Article]. 2015 2015-01-01;22(1):102-8.

29. Lasota J, Miettinen M. A new familial GIST identified. AM J SURG PATHOL. [Comment; Letter]. 2006 2006-10-01;30(10):1342.

30. Kleinbaum EP, Lazar AJF, Tamborini E, Mcauliffe JC, Sylvestre PB, Sunnenberg TD, Strong L, Chen LL, Choi H, Benjamin RS, Zhang W, Trent JC. Clinical, histopathologic, molecular and therapeutic findings in a large kindred with gastrointestinal stromal tumor. INT J CANCER. 2008 2008-01-01;122(3):711-8.

31. Brodey A, Kounnis V, Hawkes L, Jones RL, McVeigh TP, Cojocaru E. KIT-Associated Familial GIST Syndrome: Response to Tyrosine Kinase Inhibitors and Implications for Risk Management. The oncologist (Dayton, Ohio). 2022 2022-01-01;27(8):615-20.

32. Yamanoi K, Higuchi K, Kishimoto H, Nishida Y, Nakamura M, Sudoh M, Hirota S. Multiple gastrointestinal stromal tumors with novel germline c-kit gene mutation, K642T, at exon 13. HUM PATHOL. 2014 2014-01-01;45(4):884-8.

33. Wadt K, Andersen MK, Hansen TV, Gerdes AM. [A new genetic diagnosis of familiar gastrointestinal stromal tumour]. Ugeskr Laeger. [Case Reports; English Abstract; Journal Article]. 2012 2012-05-21;174(21):1462-4.

34. Bachet J, Landi B, Laurent-Puig P, Italiano A, Le Cesne A, Lévy P, Safar V, Duffaud F, Blay J, Emile J. Diagnosis, prognosis and treatment of patients with gastrointestinal stromal tumour (GIST) and germline mutation of KIT exon 13. EUR J CANCER. 2013 2013-01-01;49(11):2531-41.

35. Isozaki K, Terris B, Belghiti J, Schiffmann S, Hirota S, Vanderwinden JM. Germline-activating mutation in the kinase domain of KIT gene in familial gastrointestinal stromal tumors. AM J PATHOL. [Journal Article; Research Support, Non-U.S. Gov't]. 2000 2000-11-01;157(5):1581-5.

36. Graham J, Debiec-Rychter M, Corless CL, Reid R, Davidson R, White JD. Imatinib in the management of multiple gastrointestinal stromal tumors associated with a germline KIT K642E mutation. ARCH PATHOL LAB MED. [Case Reports; Journal Article]. 2007 2007-09-01;131(9):1393-6.

37. Vilain RE, Dudding T, Braye SG, Groombridge C, Meldrum C, Spigelman AD, Ackland S, Ashman L, Scott RJ. Can a familial gastrointestinal tumour syndrome be allelic with Waardenburg syndrome? CLIN GENET. [Case Reports; Journal Article; Research Support, Non-U.S. Gov't]. 2011 2011-06-01;79(6):554-60.

38. Engin G, Eraslan S, Kayserili H, Kapran Y, Akman H, Akyuz A, Aykan NF. Imatinib response of gastrointestinal stromal tumor patients with germline mutation on KIT exon 13: A family report. WORLD J RADIOL. [Case Reports]. 2017 2017-09-28;9(9):365-70.

39. Fornasarig M, Gasparotto D, Foltran L, Campigotto M, Lombardi S, Del SE, Buonadonna A, Puglisi F, Sulfaro S, Canzonieri V, Cannizzaro R, Maestro R. A Novel Kindred with Familial Gastrointestinal Stromal Tumors Caused by a Rare KIT Germline Mutation (N655K): Clinico-Pathological Presentation and TKI Sensitivity. J PERS MED. [Case Reports]. 2020 2020-11-17;10(4).

40. Hirota S, Nishida T, Isozaki K, Taniguchi M, Nishikawa K, Ohashi A, Takabayashi A, Obayashi T, Okuno T, Kinoshita K, Chen H, Shinomura Y, Kitamura Y. Familial gastrointestinal stromal tumors associated with dysphagia and novel type germline mutation of KIT gene. GASTROENTEROLOGY. 2002 2002-01-01;122(5):1493-9.

41. O'Riain C, Corless CL, Heinrich MC, Keegan D, Vioreanu M, Maguire D, Sheahan K. Gastrointestinal stromal tumors: insights from a new familial GIST kindred with unusual genetic and pathologic features. AM J SURG PATHOL. [Case Reports; Journal Article]. 2005 2005-12-01;29(12):1680-3.

42. Veiga I, Silva M, Vieira J, Pinto C, Pinheiro M, Torres L, Soares M, Santos L, Duarte H, Bastos AL, Coutinho C, Dinis J, Lopes C, Teixeira MR. Hereditary gastrointestinal stromal tumors sharing the KIT Exon 17 germline mutation p.Asp820Tyr develop through different cytogenetic progression pathways. GENE CHROMOSOME CANC. [Case Reports; Journal Article]. 2010 2010-02-01;49(2):91-8.

43. Arima J, Hiramatsu M, Taniguchi K, Kobayashi T, Tsunematsu I, Kagota S, Sakane J, Suzuki Y, Hirota S. Multiple gastrointestinal stromal tumors caused by a novel germline KIT gene mutation (Asp820Gly): a case report and literature review. GASTRIC CANCER. [Case Reports; Journal Article; Review]. 2020 2020-07-01;23(4):760-4.

44. Thalheimer A, Schlemmer M, Bueter M, Merkelbach-Bruse S, Schildhaus H, Buettner R, Hartung E, Thiede A, Meyer D, Fein M, Maroske J, Wardelmann E. Familial Gastrointestinal Stromal Tumors Caused by the Novel KIT Exon 17 Germline Mutation N822Y. AM J SURG PATHOL. [Case Reports; Journal Article]. 2008 2008-10-01;32(10):1560-5.

45. Ge Q, Liu Y, Yang F, Sun G, Guo J, Sun S. Chinese Pedigree with Hereditary Gastrointestinal Stromal Tumors: A Case Report and Literature Review. INT J MOL SCI. 2023 2023-01-03;24(1):830.

46. de Raedt T, Cools J, Debiec Rychter M, Brems H, Mentens N, Sciot R, Himpens J, de Wever I, Schöffski P, Marynen P, Legius E. Intestinal Neurofibromatosis Is a Subtype of Familial GIST and Results From a Dominant Activating Mutation in PDGFRA. GASTROENTEROLOGY. 2006 2006-01-01;131(6):1907-12.

47. Pasini B, Matyakhina L, Bei T, Muchow M, Boikos S, Ferrando B, Carney JA, Stratakis CA. Multiple gastrointestinal stromal and other tumors caused by platelet-derived growth factor receptor alpha gene mutations: a case associated with a germline V561D defect. J CLIN ENDOCR METAB. [Case Reports; Journal Article; Research Support, N.I.H., Intramural; Research Support, Non-U.S. Gov't]. 2007 2007-09-01;92(9):3728-32.

48. AIDAN CARNEY J, STRATAKIS CA. Stromal, Fibrous, and Fatty Gastrointestinal Tumors in a Patient With a PDGFRA Gene Mutation. The American journal of surgical pathology. 2008 2008-01-01;32(9):1412-20.

49. Ricci R, Martini M, Cenci T, Carbone A, Lanza P, Biondi A, Rindi G, Cassano A, Larghi A, Persiani R, Larocca LM. PDGFRA-mutant syndrome. MODERN PATHOL. 2015 2015-01-01;28(7):954-64.

50. Chompret A, Kannengiesser C, Barrois M, Terrier P, Dahan P, Tursz T, Lenoir GM, Bressac-De Paillerets B. PDGFRA germline mutation in a family with multiple cases of gastrointestinal stromal tumor. GASTROENTEROLOGY. 2004 2004-01-01;126(1):318-21.

51. Manley PN, Abu-Abed S, Kirsch R, Hawrysh A, Perrier N, Feilotter H, Pollett A, Riddell RH, Hookey L, Walia JS. Familial PDGFRA-mutation syndrome: somatic and gastrointestinal phenotype. HUM PATHOL. [Journal Article]. 2018 2018-06-01;76:52-7.
